# Adverse childhood experiences and profiles of healthy orthorexia versus orthorexia nervosa: towards an explanatory model of orthorexia as a multidimensional eating style

**DOI:** 10.1007/s40519-024-01694-0

**Published:** 2024-10-08

**Authors:** Marcin Rzeszutek, Joanna Kowalkowska, Małgorzata Dragan, Katarzyna Schier, Maja Lis-Turlejska, Paweł Holas, Katarzyna Drabarek, Angelika Van Hoy, Dominika Maison, Gabriela Wdowczyk, Elżbieta Litwin, Julia Wawrzyniak, Wiktoria Znamirowska, Szymon Szumiał, Małgorzata Desmond

**Affiliations:** 1 Faculty of Psychology, University of Warsaw, Warsaw, Poland; 2 Faculty of Food Science, University of Warmia and Mazury in Olsztyn, Olsztyn, Poland; 3 Faculty of Psychology, SWPS University of Social Sciences and Humanities, Warsaw, Poland; 4 Great Ormond Street Institute of Child Health, University College London, London, UK

**Keywords:** Adverse childhood experiences, Orthorexia, Alexithymia, Experiential avoidance, Embodiment, Path analysis

## Abstract

**Background:**

Orthorexia is a complex phenomenon comprising distinct dimensions, including orthorexia nervosa (ON) and healthy orthorexia (HO). However, little is known about the factors influencing these dimensions, their disparities, and the psychological factors underlying orthorexia behaviours.

**Objectives:**

This study aims to explore ON versus HO dimensions and the predictive role of adverse childhood experiences (ACEs) in a nationally representative sample of Polish individuals. In addition, we aim to investigate the mediating roles of alexithymia, embodiment, and experiential avoidance levels in this association.

**Methods:**

A representative sample of Polish adults (*n* = 3557) participated in this study. Dimensions of orthorexia (HO, ON) were assessed using the Teruel Orthorexia Scale, while ACEs were evaluated using the Adverse Childhood Experiences Questionnaire. Levels of alexithymia were measured using the Toronto Alexithymia Scale, experiential avoidance through the Acceptance and Action Questionnaire, and embodiment intensity via the Experience of Embodiment Scale.

**Results:**

Cluster analysis identified two distinctive orthorexia profiles in the whole sample, i.e., HO (*n* = 469) and ON (*n* = 1217), alongside three intermediate HO/ON profiles (*n* = 1871). The number of ACEs predicted ON tendencies as opposed to HO behaviours in participants. The mediating role of alexithymia, experiential avoidance, and embodiment in the association between ACEs and ON was also observed.

**Conclusions:**

Our study suggests that orthorexia is a multidimensional eating style shaped by socio-cultural factors. Adverse childhood experiences may be related to ON behaviours by mediating psychological factors such as experiential avoidance, alexithymia, and embodiment. Effective education and collaborative support are necessary for addressing ON tendencies.

The initial, widely accepted definition of orthorexia as an obsession with healthy and clean eating, developed by Bratman [8], initiated a long debate on the conceptualization of orthorexia as a possible new diagnostic entity (e.g., [14, 16, 29]). However, despite numerous years of intensive empirical investigation, consensus has yet to be reached regarding whether orthorexia constitutes a distinct eating disorder (ED) or a new eating style or possibly manifests as concurrent psychiatric problems with which it overlaps [5, 17]. Moreover, contemporary research has predominantly focused on the pathological aspects of orthorexia, particularly orthorexia nervosa (for reviews, see [14, 26]), a perspective criticized for stigmatizing individuals committed to healthy eating due to ideological beliefs or simply a pursuit of a wholesome lifestyle [9]. Furthermore, this unidimensional and pathological-driven approach has led to instances of over-diagnosis of orthorexia (e.g., 88.7% prevalence in non-clinical populations [18],), prompting scrutiny regarding the limitations in its measurement [28]. To address these concerns, Barrada and Roncero [6] proposed distinguishing between orthorexia nervosa (ON), characterized by a pathological obsession with adhering to a rigidly healthy diet, and healthy orthorexia (HO), denoting a broader interest in nutritious eating and a healthy lifestyle. They advocated for orthorexia to be conceptualized and assessed as a bidimensional construct. It has been observed that individuals with ON typically exhibit chronic negative affect and/or depression, poor body image, and symptoms of EDs, while those classified with HO usually lack these psychological issues and maintain a high level of well-being [15]. Nevertheless, while considerable knowledge exists about the psychosocial characteristics of ON (for reviews, see [14, 26]), research on predictors of HO remains limited (Anastasiades & Argyride [4]). Furthermore, contemporary studies on both ON and HO have predominantly employed a variable-centred paradigm, assuming that individuals with ON and HO represent distinct and homogeneous groups concerning the factors contributing to these phenomena, as well as their associated risk factors and psychosocial characteristics [14].

In our study, we adopted a person-centred approach to examine whether participants could be classified into versatile ON or HO profiles exhibiting heterogeneous associations with the variables under investigation, aiming to gain a deeper understanding of the disparities between the pathological and healthy dimensions of orthorexia [47]. Specifically, we investigated the association between the number of adverse childhood experiences (ACEs), as defined as negative or potentially traumatic life events that occur during childhood and adolescence [19], as predictors of ON versus HO in a national sample of Poles.

Numerous studies have shown strong associations between childhood trauma and adversity exposure and the susceptibility to various EDs in adulthood (for reviews and meta-analyses, see [13, 44]). However, upon closer examination of these findings, it becomes apparent that several research gaps still need to be addressed [20, 44]. First, the relationships between childhood trauma and diverse ED types exhibit considerable heterogeneity, displaying strong and positive correlations with binge eating disorder and obesity [44], inconclusive evidence regarding anorexia or bulimia [13] and, to the best of our knowledge, only two studies devoted to orthorexia [22, 27]. Second, the vast majority of authors have focused mainly on physical and sexual abuse, with very few studies devoted to other types of traumatic experiences in childhood, such as emotional abuse and neglect [13]. Third, there is still no convincing mechanism linking childhood trauma and ED. Attempts to find some direct genetic or epigenetic explanations have failed [21]. A more promising avenue involves exploring the indirect relationship between childhood trauma history and the risk of diverse EDs in adulthood, such as emotion dysregulation [36], distorted body image (Malecki et al., 2018, or the co-occurring symptoms of other psychiatric disorders, e.g., depression or obsessive–compulsive disorder [OCD] [23, 47]).

Therefore, in our study, we explored the relationship between the number of ACEs, defined as negative or potentially traumatic life events that occur during childhood or adolescence [19], and profiles of ON and HO among participants. In addition, we examined the mediating role of alexithymia, embodiment, and experiential avoidance in potentially underpinning this association concerning orthorectic tendencies in our sample (Anastasiades & Argyride, 2022; [30]). Multiple studies have shown various problems with emotion regulation among people suffering from ED [24], particularly alexithymia, a neuropsychological syndrome characterized by significant obstacles in recognizing, expressing, and describing one’s emotions [43]. However, concerning orthorexia, only one study demonstrated that higher levels of alexithymia, along with emotional dysregulation, predicted more pronounced tendencies towards ON [30]. In addition, while a substantial body of literature has underscored the importance of negative body image as a mechanism contributing to the maintenance of EDs [35], only one study has examined the role of positive body image concerning levels of ON and HO (Anastasiades & Argyride, 2022). In our research, we focused on the broad construct of embodiment, which that captures the quality of the experiences of living in the body, from positive to negative embodiment, describing body connection and comfort, agency and functionality, attuned self-care, bodily desires, and freedom from objectification [35].

Finally, experiencing traumatic events can be linked to experiential avoidance, which refers to the tendency of individuals to avoid or suppress uncomfortable thoughts, emotions, sensations, or memories. Specifically, trauma can lead to the development of experiential avoidance as a coping mechanism to avoid distressing reminders of a traumatic event [33]. Individuals may engage in behaviours such as substance abuse or disordered eating patterns as a means to avoid confronting the painful memories or emotions associated with the trauma [33]. Moreover, some authors conceptualize orthorexia as a spectrum that encompasses symptoms of ED (anorexia and bulimia) alongside symptoms of OCD [34]. All these disorders share certain cognitive features in common, particularly experiential avoidance, which is operationalized by low levels of psychological flexibility [34]. A recent review showed that high experiential avoidance is a key cognitive element sustaining ED symptoms and precluding successful psychotherapy among individuals suffering from various types of ED [31].

## Current study

Given the existing research gaps in the contemporary literature on orthorexia, our study aimed to address three main objectives. First, employing a person-centred approach, we sought to identify profiles of HO and ON, including intermediate HO/ON subgroups, within a large non-clinical sample of Polish individuals, thereby potentially confirming the multidimensional structure of orthorexia. Second, we aimed to explore the relationship between the number of ACEs and the intensity of HO versus ON tendencies among our participants, while considering the potential mediating roles of alexithymia, experiential avoidance, and embodiment. Finally, we aimed to control our findings for various sociodemographic characteristics and the prevalence of obesity (BMI ≥ 30 kg/m^2^). We hypothesized that our sample would exhibit heterogeneity regarding orthorectic tendencies, encompassing individuals with varying degrees of both ON and HO (Hypothesis 1). In addition, we assumed that the number of ACEs would positively correlate with ON intensity and negatively correlate with HO intensity among participants (Hypothesis 2). Lastly, we expected that higher levels of alexithymia, lower levels of embodiment, and higher levels of experiential avoidance would be associated with a greater likelihood of developing ON compared to HO (Hypothesis 3). Overall, our goal was to construct an explanatory and theory-driven model of orthorexia, which could enhance understanding of the psychological foundations of orthorexia and aid clinicians in distinguishing individuals with disordered eating tendencies (ON) from those with health-conscious eating patterns (HO).

## Methods

### Participants and procedure

In January and February 2024, an external survey-specialized company facilitated the data collection process for a nationwide research panel in Poland. The study questionnaires were distributed to the participants via an online panel. Participation was voluntary and anonymous, with all participants providing informed consent. Participants were incentivized with tokens exchangeable for money. The project received approval from the Ethics Committee of the Faculty of Psychology, University of Warsaw, Poland.

**Fig. 1:**
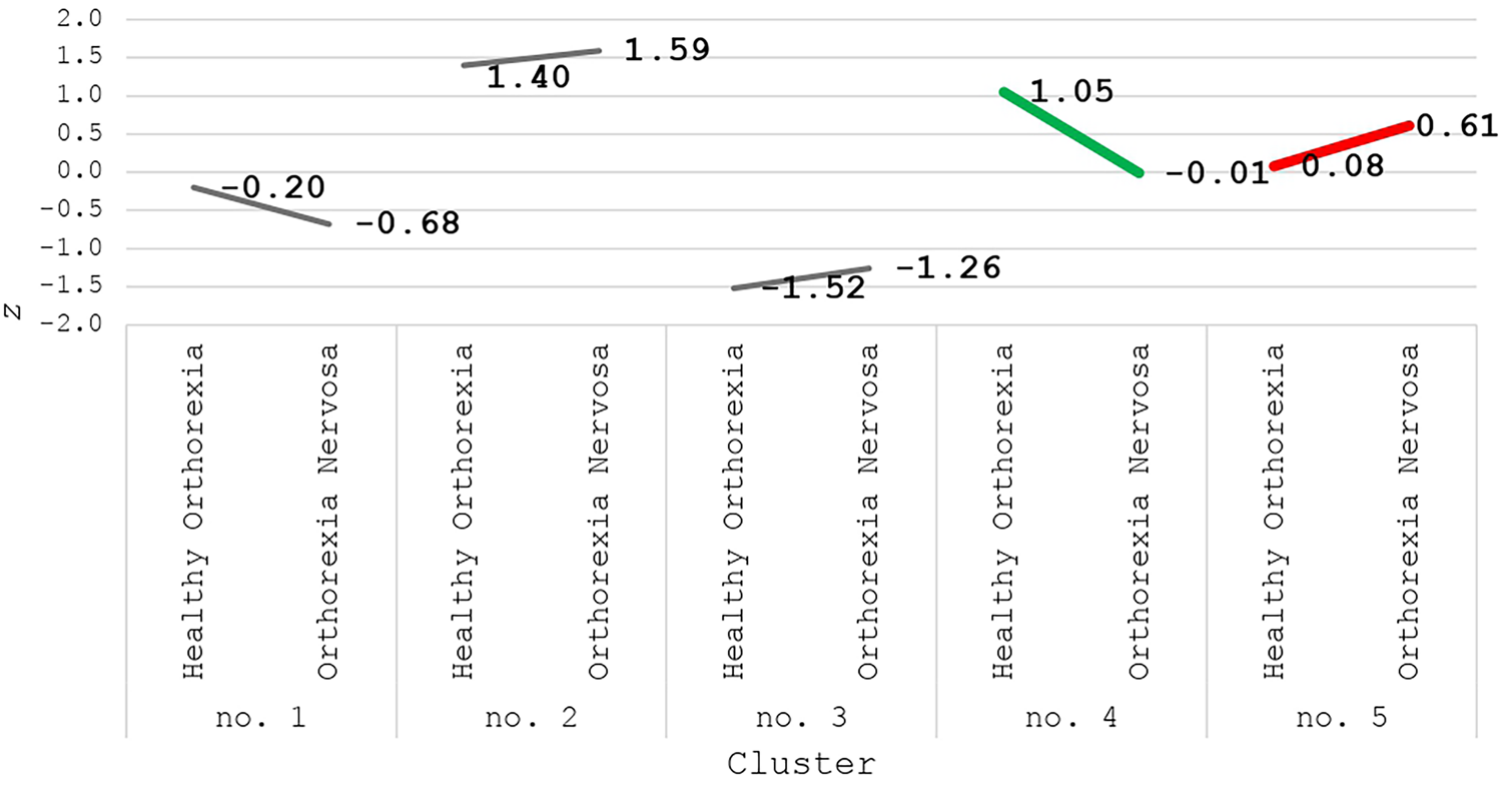
Mean values of standardized scores in extracted clusters of healthy orthorexia and orthorexia nervosa in the whole study sample (*n* = 3557)

The initial study sample comprised 3557 participants aged 18–99. For the final analysis, only individuals displaying clear indicators of HO and ON were included. The final sample (n = 1686) was selected through cluster analysis based on data collected utilizing the Teruel Orthorexia Scale (TOS), which identified five clusters with various levels of HO and ON (see Measures; Fig. 1, and for details). The remaining 1871 participants, representing heterogeneous profiles of both HO and ON (Fig. 1), were not included in the final path model but were utilized to verify the first hypothesis of the study (Table 1 and Fig. 1).

**Table 1:** Sociodemographic variables in the final sample (*n* = 1686)

	Orthorexia					
	Healthy	Nervosa				
Variable	n (469)	n (1217)	χ^2^/t	df	p	V/d
Gender						
Female	299 (63.8%)	624 (51.3%)	21.28	1	0.001	0.11
Male	170 (36.2%)	593 (48.7%)				
Age in years (M ± SD)	51.52 ± 15.09	44.40 ± 15.70	8.44	1684	0.001	0.46
Place of residence						
Village	151 (32.2%)a	449 (36.9%)^a^	9.78	4	.044	.04
Small town: up to 20 thousand residents	52 (11.1%)a	173 (14.2%)a				
City: 20 to 99 thousand residents	117 (24.9%)a	246 (20.2%)b				
City: 100 to 500 thousand residents	92 (19.6%)a	203 (16.7%)a				
City: over 500 thousand residents	57 (12.2%)a	146 (12.0%)a				
Education						
Elementary	3 (.6%)a	41 (3.4%)b	35.13	6	0.001	0.14
Vocational	32 (6.8%)a	129 (10.6%)b				
Secondary	176 (37.5%)a	501 (41.2%)a				
Higher education: BA	36 (7.7%)a	117 (9.6%)a				
Higher education: engineering	19 (4.1%)a	58 (4.8%)a				
Higher education: MA	199 (42.4%)a	358 (29.4%)b				
PhD and over	4 (.9%)a	13 (1.1%)a				
Employment						
Odd job	20 (4.3%)a	100 (8.2%)b	39.12	4	0.001	0.15
Full employment	253 (53.9%)a	713 (58.6%)a				
Retired or illness allowance	158 (33.7%)a	250 (20.5%)b				
Unemployed/parental leave/house running	25 (5.3%)a	95 (7.8%)a				
No, studying	13 (2.8%)a	59 (4.8%)a				
Financial situation on a scale of 1–7 (M ± SD)	4.71 ± 1.21	4.43 ± 1.19	4.34	1684	0.001	0.24
Obesity, BMI ≥ 30 kg/m^2^	124 (26.4%)	597 (49.1%)	70.74	1	0.001	0.21

*M* mean, *SD* standard deviation, *χ*^*2*^ Pearson’s χ^2^ test for independence, *t* independent samples *t* test, *df* degrees of freedom, *p* statistical significance, *V* Cramer’s V effect size measure, *d* Cohen’s d effect size measure, a, b – different letters mark statistically significant differences on the level based on Bonferroni correction

The extracted subgroups HO and ON differed in every sociodemographic variable. These variables were analysed as controlled variables in further analysis.

## Measures

### Orthorexia

The Polish adaptation of the TOS, developed by Barrada and Roncero [6], was utilized in this study to assess the levels of ON and HO among participants. The TOS consists of 17 items and evaluates orthorectic eating behaviours across two proposed dimensions: HO (9 items) and ON (8 items). Responses to TOS items were rated on a 4-point Likert scale ranging from 0 (completely disagree) to 3 (strongly agree). The Polish version of the TOS has been adapted and validated for use in the Polish population by Styk et al. [41], ensuring its applicability and validity in this context. Higher scores on both dimensions indicate a higher presence of HO versus NO. The Cronbach’s alphas for both ON and HO were satisfactory and are presented in Table 2.

**Table 2:** Descriptive statistics and Pearson correlation coefficients between analysed variables

Variables	Descriptive statistics					Pearson correlation coefficient				
	*M*	*SD*	*S*	*K*	α	1	2	3	4	5
1. 1. No. of adverse childhood experiences	1.53	2.04	1.57	2.20	0.78	–	–	–	–	–
1. 2. Embodiment	118.72	20.19	0.07	−0.15	0.94	−0.243**	–	–	–	–
2. 3. Alexithymia	53.44	12.07	−0.41	−0.35	0.88	0.177**	−0.687**	–	–	–
3. 4. Experiential avoidance	22.02	10.32	0.23	− .63	0.95	0.338**	−0.649**	0.647**	–	–
4. 5. Healthy orthorexia	26.52	7.42	−0.19	0.02	0.91	−0.071**	0.144**	0.041*	0.035*	–
5. 6. Orthorexia nervosa	19.78	6.90	0.10	−0.40	0.90	0.019	−0.325**	0.422**	0.321**	0.676**

*M* mean value, *SD* standard deviation, *S* skewness, *K* kurtosis, α Cronbach’s α reliability coefficient, * *p* < .05; ** *p* < .01

### Adverse childhood experiences

The Polish version of the Adverse Childhood Experience Questionnaire was used in this study [19]. ACE-10 is a self-report tool designed to assess retrospective childhood traumatic or adverse experiences. It includes 10 items that cover two categories of experiences: five types of maltreatment (physical abuse, emotional abuse, sexual abuse, physical neglect, and emotional neglect) and five types of household dysfunction (parental separation/divorce, household physical violence, household substance abuse, household mental illness or suicide attempt, and incarcerated household members). Respondents answer these questions with a yes or no response. The ACE-10 calculates a cumulative score by summing the number of “yes” responses, providing a severity index ranging from 0 to 10, which indicates the number of different adversities experienced in childhood. The Cronbach’s alphas for this scale were satisfactory and are presented in Table 2.

### Alexithymia

The Polish adaptation of the Toronto Alexithymia Scale (TAS-20; [3]) was used in this study. TAS-20 is a 20-item self-report tool. The items are grouped into three subscales: (1) Difficulty Identifying Feelings (DIF), which assesses the ability to identify and distinguish between feelings and bodily sensations during emotional arousal (seven items, e.g., “I have feelings that I can’t quite identify”), (2) Difficulty Describing Feelings (DDF), which evaluates the ease of describing feelings (five items, e.g., “People tell me to describe my feelings more”), and (3) Externally Oriented Thinking (EOT), which assesses a tendency to focus on external events rather than internal emotional experiences (eight items, e.g., “I prefer to analyse problems rather than just describe them”). Each item is rated on a 5-point Likert scale, ranging from 1 (strongly agree) to 5 (strongly disagree). In this study, the total scores on alexithymia were analysed among participants. Cronbach’s alphas for the total alexithymia scale were satisfactory and are presented in Table 2.

### Experiential avoidance

To evaluate the level of experiential avoidance in our sample, the Polish adaptation of the Acceptance and Action Questionnaire—version II (AAQ-II; [7]) was used. This tool defines psychological flexibility as the ability to fully contact with the current experienced psychological states (i.e., thoughts, feelings, and physiological sensations) without the need for defences over them or engaging in behaviours to modify them. In contrast, psychological inflexibility, as a sign of experiential avoidance, is a rigid attempt to alter the form, frequency, or situational sensitivity of the above-mentioned inner experiences, even when doing so may lead to behavioural difficulties. Higher total scores on the AAQ-II indicate higher psychological inflexibility (experiential avoidance). In contrast, lower total scores indicate more psychological flexibility. The Cronbach’s alphas for the experiential avoidance scale were satisfactory and are presented in Table 2.

### Embodiment

The Polish adaptation of the Experience of Embodiment Scale (EES), developed by Piran et al. [35], served as a tool to gauge embodiment in this study. This scale explores the nuanced aspects of individuals’ embodied experiences, encompassing six distinct dimensions. These include Body Connection and Comfort, which explores feelings of harmony with one’s body, Body-Unencumbered Adjustment, which assesses the impact of body dissatisfaction on social interactions (reverse scored); Agency and Functionality, focusing on the comfort in expressing personal views and beliefs; Experience and Expression of Sexual Desire, which evaluates the ability to communicate sexual desires effectively; Attuned Self-Care, examining the extent of care and the respect shown towards one’s body; and Resisting Objectification, which emphasizes valuing the body’s capabilities over its appearance. Participants rated their agreement with EES items on a 5-point scale ranging from 1 (strongly disagree) to 5 (strongly agree), with reverse scoring applied to negative items. In this study, total embodiment scores were computed to reflect the overall experience of embodiment, with higher scores indicating a more positive embodiment experience. The Cronbach’s alphas for the total embodiment scale were satisfactory and are presented in Table 2.

### Data analysis

First, the groups of participants with HO versus ON were extracted with the use of cluster analysis, which aimed to cluster study participants in such a way that individuals in the same group (in a cluster) are more similar (in some specific way) to each other than to those in other groups [2]. The extracted groups were then verified regarding possible associations with sociodemographic variables with the use of the Pearson chi-square test of independence and Student’s *t* test. Differences regarding analysed interval variables were verified with the use of Student’s *t* test for independent samples. Cramer’s V and Cohen’s d effect size measures were provided for categorical and interval variables, respectively. The higher the value of each effect size measure, the higher the difference between the groups. The sociodemographic variables with a consistent pattern of relationships with the two dimensions of orthorexia were included in the preliminary model of relationships between the analysed variables. The sociodemographic variables and the dimensions of orthorexia were categorical variables. Therefore, in the next step, the Bayesian approach to path analysis was used in further analysis [12]. The Bayesian approach to path analysis allows for mixing both interval and categorical variables in a single model of relationships between variables based on previous research and theory as its starting point. The group with HO was extracted in the course of the cluster analysis and was analysed as a reference group. In the course of analysis, each relationship and its path acquire a credible interval. To verify Hypothesis 1, the credible interval for an indirect effect was also calculated.

## Results

### Descriptive statistics

Table 2 depicts the descriptive statistics and Pearson’s correlation coefficients.

### Profiles of orthorexia

The standardized scores acquired on the HO scale and the ON scale were analysed with cluster analysis to extract subgroups with distinctive profiles of orthorexia. We decided to use this method to verify whether exclusive groups of ON and HO could be extracted without preliminary numerical assumptions, just on the basis of the scores of respondents in TOS (see Measures). Figure 1 presents the final cluster centres, i.e., the mean values of standardized scores in each cluster extracted.

In cluster no. 1 (*n* = 797), HO and ON did not differ from each other, and the level of HO was very close to the average value. In cluster no. 2 (*n* = 408), the level of both HO and ON was high. In cluster no. 3 (*n* = 666), the level of HO and ON was low. In cluster no. 4 (*n* = 469) the level of HO was high, while the level of ON was average. This group was recognized as a group of participants with HO (Fig. 1—the green line). In cluster no. 5 (*n* = 1217), the level of ON was high, while the level of HO was average. This group was recognized as the group of participants with ON (Fig. 1—the red line).

### Between-group comparisons

Table 3 depicts the mean values of analysed interval variables in the extracted groups of participants with HO and participants with ON with the values of Student’s *t* test for independent samples.

**Table 3:** Mean values of analysed variables in the extracted subgroups

	Orthorexia							
	Healthy		Nervosa					
Variables	*M*	*SD*	*M*	*SD*	*t*	*df*	*p*	*d*
No of adverse childhood experiences	1.29	1.82	1.51	2.10	− 2.10	972.51	0.018	−0.11
Embodiment	132.12	16.97	109.39	15.78	25.13	797.66	0.001	1.41
Alexithymia	47.75	12.37	58.55	8.15	− 20.91	631.06	0.001	− 1.14
Experiential avoidance	18.25	9.56	24.97	8.77	− 13.74	789.20	0.001	0.75

*M* mean value, *SD* standard deviation, *t* independent samples *t* test, *df* degrees of freedom, *p* statistical significance, d Cohen’s d effect size measure

The mean values of the number of ACEs, levels of alexithymia, and experiential avoidance were significantly higher in the group of participants with ON compared to HO, while the mean value regarding the level of embodiment was significantly lower among ON compared to the HO group.

### Path model of relationships between analysed variables

**Fig. 2:**
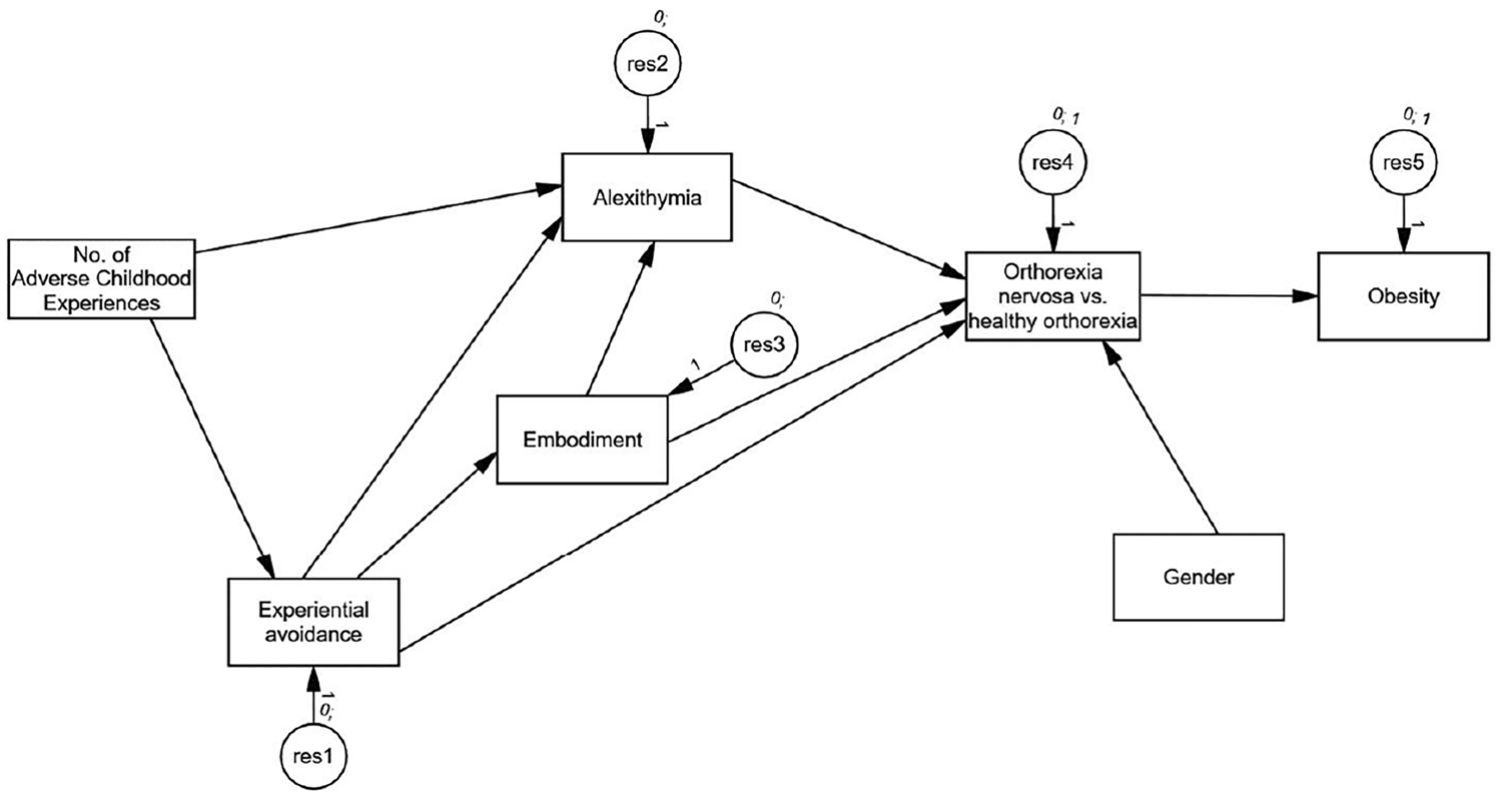
Path model of the relationship between analysed variables among participants from the orthorexia nervosa cluster (*n* = 1217) compared to participate from the healthy orthorexia cluster (*n* = 469

Figure 2 depicts the verified model of the relationships between variables. ON was the analysed categorical variable. HO served as the baseline comparison. Sociodemographic data were analysed as controlled variables. As the credible intervals for regression coefficients for participants’ age [− 0.01; 0.01], education [− 0.26; 0.04], and financial situation [− 0.11; 0.02] all encompassed zero, these variables were excluded from the model. Table 4 depicts credible intervals for the associations between the analysed variables obtained in the model. The convergence statistic for the final model was *Conv* = 1.001.

**Table 4:** Credible intervals for regression coefficients acquired in the final model

Relationships			Credible interval
Alexithymia	< –	No. of adverse childhood experiences	[−0.69; − 0.40]
Experiential avoidance	< –	No. of adverse childhood experiences	[1.21; 1.63]
Embodiment	< –	Experiential avoidance	[− 1.38; − 1.24]
Alexithymia	< –	Experiential avoidance	[0.29; 0.39]
Orthorexia	< –	Experiential avoidance	[− 0.02; − 0.01]
Alexithymia	< –	Embodiment	[− 0.32; − 0.27]
Orthorexia	< –	Embodiment	[− 0.05; − 0.04]
Orthorexia	< –	Alexithymia	[0.02; 0.04]
Orthorexia	< –	Gender	[0.23; 0.53]
Obesity	< –	Orthorexia	[0.35; 0.48]

The relationship between the number of ACEs and ON was mediated by the intensity of alexithymia, embodiment, and experiential avoidance [0.06; 0.10]. Namely, the higher the number of ACEs, the higher the level of alexithymia and, parallelly, the higher the level of experiential avoidance, which in turn increased the intensity of ON tendencies in participants. In addition, a higher level of experiential avoidance was negatively related to the levels of embodiment, and this lower level of embodiment reinforced the intensity of alexithymia among participants, which reciprocally increased the likelihood of developing ON in contrast to HO. We also found that the intensity of ON tendencies was positively associated with the prevalence of obesity, as measured by BMI in our sample. Finally, in contrast to HO, the intensity of ON tendencies was higher among men than among women.

## Discussion

Our findings suggest that orthorexia can be a multidimensional eating style. We extracted two profiles of orthorexia among adults, namely HO and ON, but we also observed several intermediate HO/ON subgroups. Although Barrada and Roncero [6] proposed some time ago to conceptualize orthorexia as a bidimensional eating style, highlighting both its healthy and pathological aspects, this hypothesis has yet to be empirically validated. Specifically, the person-centred approach has not been utilized in data analysis to investigate this conceptualization. To the best of our knowledge, only Yakin et al. [47], employing a typological approach, identified naturally distinct groups representing HO versus ON behaviours in a non-clinical population. However, they also noted participants who fell “in-between” these subgroups. This suggests that orthorexia may not necessarily be defined as bidimensional, but rather as a multidimensional eating style. Although HO and ON are two theoretically distinct but overlapping eating styles, it is possible for them to coexist within the same individual. In this study, following a person-centred approach, we found five orthorexia profiles—two of them were characterized by high intensity of HO or ON tendencies. Therefore, our findings may shed not only new light on the nature of orthorexia and its dimensions [5, 28] but also facilitate clinicians in better identifying and selecting individuals with ON tendencies versus those who follow healthy eating for health or ideological reasons [9]. Furthermore, the “in-between” HO/ON profiles can serve as a starting point for early prevention of potential ON tendencies. However, further prospective studies are needed to investigate the dynamics of such profiles.

Although the literature on psychosocial characteristics of orthorexia is vast (for a review, see [26]), there is a lack of research on psychological mechanisms potentially leading to this syndrome. In this study, we focused on ACEs, which are a transdiagnostic risk factor for psychopathology, through the impact on stress response systems leading to neurobiological alterations associated with executive, attentional, and affective emotional dysregulation [39]. It was found that the number of ACEs was positively associated with the intensity of ON tendencies and negatively linked to the intensity of HO among participants. Although numerous studies have shown the long-term effects of childhood trauma on the risk of EDs in adulthood (for reviews and meta-analyses, see [13, 44]), similar research with respect to orthorexia is very scarce [22, 27]. Guillaume et al. [20] claim that the possible pathway linking exposure to ACEs and the risk of ED in adults lies in trauma-related problems with emotion regulation. These difficulties may have a reciprocal impact on distorted cognitive functioning and interpersonal problems among ED patients [32].

In our study, we investigated the mediating role of alexithymia, experiential avoidance, and embodiment in the association between ACEs and orthorexia, which corresponded to our last hypothesis. We observed that a higher number of ACEs had two separate consequences: a higher level of alexithymia and a higher intensity of experiential avoidance in participants, which in turn led to greater ON tendencies in contrast to HO behaviours. In addition, a higher level of experiential avoidance was negatively related to the levels of embodiment, and this lower level of embodiment reinforced the intensity of alexithymia among participants, which reciprocally also increased the likelihood of developing ON compared to HO. The link between traumatic events and experiential avoidance is supported by research, indicating that trauma can lead individuals to engage in experiential avoidance as a coping strategy [33]. Similarly, experiential avoidance is connected to EDs, suggesting that individuals may adopt disordered eating behaviours as a way to avoid or suppress their emotions. Previous studies also showed that ED patients suffer from various emotion recognition deficits, particularly alexithymic tendencies [10]. Shank et al. [38] argued that these tendencies forced such patients to engage in emotional eating or even binge eating. Recently, Obeid et al. [30] underscored that in the case of people with ON, stating that obsessive rituals towards a healthy and clean diet may act as a similar way to take some control over negative or destructive emotions, which are not perceived by them consciously due to alexithymic patterns. Moreover, some authors treat orthorexia as a potential spectrum disorder combining the characteristic features of some EDs, e.g., anorexia and bulimia, with features of OCD, which are also closely linked with experiences of childhood trauma [34]. The pathological side of orthorexia (ON) has its source in symptoms overlapping with OCD, such as recurrent, intrusive thoughts, fixations (similar to obsessions) about food, its potential contamination, and impurities, which cause anxiety and impede the functioning of the individual, resulting in a strong need for the preparation and consumption of certain foods. Orthorexia, anorexia, and OCD are characterized by high cognitive rigidity, anxiety, and experiential avoidance [34]. Experiential avoidance (or low psychological flexibility) is also considered crucial to understanding compulsive behaviour as a short-term escape from negative emotions. This is the key mechanism for maintaining an obsession with a rigidly healthy diet in orthorexia [30]. However, in our model, we observed one more interesting feature linked to this variable, namely that experiential avoidance decreases the level of embodiment in participants, which indirectly was positively associated with ON tendencies. Several directions of research suggest that experiential avoidance is likely to have a negative impact on the level of embodiment. First, avoidance behaviours can disrupt the connection between an individual and their bodily experiences, leading to a disconnection from physical sensations and experiences. In addition, mindfulness and embodiment interventions are used to counter experiential avoidance and enhance the capacity for embodiment, indicating that experiential avoidance can hinder embodiment. To the best of our knowledge, only Anastasiades and Argyrides [1] have explored the role of embodiment in individuals with ON and HO, noting a significantly lower level of embodiment in the ON group compared to the HO subsample. In our study, we observed a similar trend, with the additional finding of a potential explanation. Individuals with ON displayed lower psychological flexibility compared to those with healthy orthorexia. Psychological flexibility is also positively associated with intuitive eating, characterized by eating in response to pure physiological hunger rather than to fulfil emotional needs [37]. Intuitive eating is closely linked with facets describing embodiment [35] and may, therefore, be considered the opposite of ON and a feature characterizing healthy orthorexia, as observed in the study by Anastasiades and Argyrides [1].

Finally, we noticed that ON tendencies were higher among males than females and were positively linked with the prevalence of obesity (BMI ≥ 30 kg/m^2^). Research on gender differences in ON provides mixed findings, with studies revealing higher ON levels in women, higher levels in men than women, or a lack of significant differences in this regard [40]. Nevertheless, there is greater consensus on the positive relationship between ON and BMI [11, 15], which may be a sign that ON, despite apparent care for the healthiness of diet, is in fact the opposite of a healthy lifestyle and in its obsessive, pathological side may even be a risk factor of obesity [30].

### Strengths and limitations

This study has several strengths, including its large and representative sample size, theory-driven and person-centred approach to the study of both dimensions of orthorexia, and the distinctive constellation of study variables aimed at elucidating the mechanisms of this multidimensional eating style. However, due to organizational constraints, we encountered some limitations. First, the study was conducted online by an external company, potentially overlooking individuals who are still digitally excluded. Second, although we employed valid and psychometrically sound measures, the self-report nature of the assessment may introduce social desirability bias into the collected data and obtained findings. Finally, despite utilizing path analysis to explore potential causal relationships between variables [12], the cross-sectional design of our research precludes making causal inferences. The findings of this study require verification in a prospective research to examine whether HO and ON profiles remain stable over time or whether participants from these profiles may shift between subgroups.

## Conclusion

Our study sheds new light on the theoretical understanding of orthorexia and its measurement, with potential clinical implications. Our findings suggest that orthorexia can be understood as a multidimensional eating style, influenced by contemporary socio-cultural phenomena, particularly exacerbated by misinformation propagated through social media regarding what constitutes a healthy versus pathological eating style [42]. Apart from a pathological obsession with healthy and clean eating (ON), some people may generally be interested in healthy food and lifestyles (HO). However, orthorexia should not be narrowly construed as a bidimensional construct; rather, it encompasses a multidimensional spectrum where individuals may fall “in-between” HO and ON. Given the limited research on the psychological mechanisms underlying orthorexia, our study examined the role of ACEs and identified the mediating effects of alexithymia, experiential avoidance, and embodiment in the association between ACEs and orthorexia. Effective education on healthy eating and strategies to cope with negative emotions resulting from ACEs are essential in preventing ON tendencies in individuals predisposed to the pathological aspects of orthorexia. Our findings underscore the importance of collaboration between clinical psychologists and dieticians to accurately identify individuals with ON tendencies and those who pursue healthy eating for health or ideological reasons.

## Data Availability

No data sets were generated or analysed during the current study.
